# The evolutionarily stable strategy, animal contests, parasitoids, pest control and sociality

**DOI:** 10.1098/rstb.2021.0498

**Published:** 2023-05-08

**Authors:** Ian C. W. Hardy, Mike Mesterton-Gibbons

**Affiliations:** ^1^ Department of Agricultural Sciences, University of Helsinki, PO Box 27, FI-00014 Helsinki, Finland; ^2^ Department of Mathematics, Florida State University, 1017 Academic Way, Tallahassee, FL 32306-4510, USA

**Keywords:** game-theoretic models, dyadic contests, parasitoids, life histories, biocontrol, sociality

## Abstract

The evolutionarily stable strategy, ESS, concept was first used in biology to understand sex ratio bias and, shortly afterwards, to explore the logic of contests over essential and indivisible resources. ESS models formed the basis of much subsequent research on animal behaviour and placed game-theoretic thinking firmly within the behavioural ecology approach. Among behavioural ecologists studying parasitoids, it was those asking questions about the evolution of sex ratios who first made extensive use of the game-theoretic approach. A later growth of interest in parasitoid host defence and fighting behaviour made use of these tractable study species to explore contests and their connections to further aspects of life-history evolution plus some pest control applications. Our aims are to (i) introduce the topic of contests, which are engaged in by a very wide array of animal taxa, and the importance, both historical and conceptual, of the game-theoretic approach to their study, and (ii) review recent studies of parasitoid contests, including those that have considered the context of social evolution and the performance of parasitoids as agents of biological control. We consider that game-theoretic models are eminently testable and applicable and will likely endure as valuable tools in studies of parasitoid biology.

This article is part of the theme issue ‘Half a century of evolutionary games: a synthesis of theory, application and future directions’.

## 1. Introduction

The concept of the evolutionarily stable strategy, ESS, has its origins in the game theory developed in behavioural economics [1,2]. A key difference is that in human behavioural economics, rational forethought provides the means of arriving at an optimal behavioural strategy while, for most organisms, rational forethought is best assumed absent and the blind watchmaker of evolutionary natural selection [3] provides the optimizing agent that, over many generations, leads to individuals expressing optimal strategies in the presence of competing decision makers.

It is well known that the first explicitly game-theoretic model in evolutionary biology was William D. Hamilton's theory of local mate competition, which used the term ‘unbeatable strategy’ to capture the essence of an organism's best-evolved response [4,5]. This theory was stimulated by, and has broadly explained, the extremely biased sex ratios observed in many invertebrate species, including parasitoid wasps [6–9]. Nevertheless, earlier biologists had used game-theoretic reasoning either implicitly (e.g. [10]) or with species rather than individuals as players [11].

It is equally well known that the term ‘ESS’ was first used in an initially separate strand of behavioural ecology research, sparked by John Maynard Smith and George Price, who constructed models to understand the logic of animal contests over essential and indivisible resources [12–14]. These models formed the basis of a tremendous amount of subsequent research into contests, and many other aspects of animal behaviour, and placed game theory firmly within the behavioural ecologist's conceptual tool kit [15,16]. Our first aim in this article is to introduce the topic of contests, which are engaged in by a very wide array of animal taxa, and the importance, both historical and conceptual, of the game-theoretic approach to their study. We note that several reviews published around a decade ago captured the key material in some detail [17–19], and we thus provide here only a brief summary, highlighting recent studies.

The adults of most species of parasitoid wasps are not known to engage in dyadic contests over resources, and the initial surge of contest research proceeded for several decades with little reference to parasitoids. During the same period, parasitoids were proving to be among the key organisms in the expansion of sex ratio theory and in many other areas of behavioural and ecological research [6,7,20]. Around 25 years ago, researchers started to take more note of the fact that some species of parasitoids do engage in direct contests over resources. The framework developed from Maynard Smith and Price's impetus was readily adopted to structure the subsequent empirical research. The list of parasitoid species exhibiting contest behaviour grew as new interest in this area of their life histories was generated. Research on parasitoid contests has now provided some of the more comprehensive sets of studies (both empirical and theoretical) of the factors that determine the durations, outcomes and consequences of animal contests and has generated insights into other areas of parasitoid life histories (including clutch size and sex ratio decisions) that can be affected by contest behaviour. As these developments were also reviewed around a decade ago [21], our second aim in this article is to focus on more recent studies of the behavioural ecology of parasitoid contests and associated life-history phenomena, including cooperation and conflicts during multi-mother reproduction and how such contest theory has been used to explore possible trajectories of biological pest control performance when parasitoids are faced with exotic host species.

## 2. The concept of contests

A contest is a strategic interaction in which two or more individuals compete for a prize (a ‘resource’). There is an extensive literature on contest theory in both economics and behavioural ecology, but these two literatures have different goals and have developed in very different directions. Broadly speaking, economists study community games, i.e. games whose players are specific actors [2,22], whereas behavioural ecologists study population games, i.e. games whose players are individuals drawn randomly from a large population [12,15,19]. In economics, where contest theory has a quite restricted formalism, strategies are typically continuous levels of effort expended in various ways to outcompete rivals [23,24]: the focal interaction is between specific actors for a specific prize. By contrast, in behavioural ecology, the focal interaction is a representative contest between individuals drawn randomly from a large population and contest theory is much more expansive [17,18,25]. It allows several different kinds of strategy in addition to levels of effort, for example, aggression thresholds, proportions, probabilities and continuous approximations of discrete quantities. Discrete strategies are also used.

Continuous approximation of a discrete quantity is exemplified by Mesterton-Gibbons & Hardy [26]: this study analyses the effect of body size-dependent contest outcomes on optimal clutch size, especially in a parasitoid (members of the bethylid genus *Goniozus*). Threshold strategies are exemplified by Mesterton-Gibbons *et al*. [27]: this study explores whether volatile chemical (spiroacetal) emissions can serve as a weapon of rearguard action, especially in *Goniozus*. Discrete strategies are exemplified by Mesterton-Gibbons *et al*. [28], which explores whether size advantage can sustain a maternal preference to use a more deadly host species, again, especially in a parasitoid (members of the scelionid genus *Trissolcus*). We focus on these examples because all three illustrate both the expansiveness of contest theory within behavioural ecology and the extent to which game theory has proved productive in the study of parasitoid behaviour. For examples of the other strategies, see Mesterton-Gibbons [16].

Although game-theoretic models can be tailored to the biological details of a given species [29,30], they are typically most useful as general models that allow us to test the logic of a verbal argument rigorously. In that context, it need not matter that an ESS fails to be directly observable (for example, if an animal is being aggressive, is it using a strategy of obligate aggression, or is its aggression conditional on other, unmeasured, factors?). Does it matter in the context of testing observed behaviour? The answer is, fortunately, much less than might appear, simply because the probabilities of outcomes associated with an ESS can be compared with observed frequencies. For example, Mesterton-Gibbons *et al*. [27] calculate the probability of volatile chemical release at the ESS as a function of their model parameters and are able to compare it with an observed value in *Goniozus*. The prediction matches the observation for a plausible range of model parameters. Although these parameters remain unobserved, the important point is that whether an ESS is testable is ultimately more of an empirical than a theoretical issue.

## 3. Parasitoid contests and contexts

Parasitoids are free-living insects as adults but their offspring feed on a single host, normally another insect, which is invariably killed [6]. The majority of parasitoid species belong to the order Hymenoptera and only these taxa, known as ‘parasitoid wasps’, will be considered here. Most belong to the Parasitica (e.g. the families Encyrtidae, Eulophidae, Eupelmidae, Ichneumonidae and Scelionidae), but some, such as the family Bethylidae, belong to the Aculeata, which also contains the ants, bees and wasps that have evolved ‘advanced’ sociality (eusociality). Parasitoid aculeates are typically socially solitary but some have evolved social behaviours, such as maternal care (sub-sociality) for the developing brood (e.g. the bethylid genus *Goniozus*) and, in a few cases, cooperative brood care (quasi-sociality) by multiple contributing mothers (e.g. the bethylid genus *Sclerodermus*). Some species in the Parasitica also exhibit aspects of sociality, such as reproductive division of labour (non-reproductive soldier castes among the reproductive larvae in some clonally developing encyrtids, [31,32]) and communal brooding, but without cooperative brood care, in the eulophid genus *Melittobia* [33].

**Figure 1.**
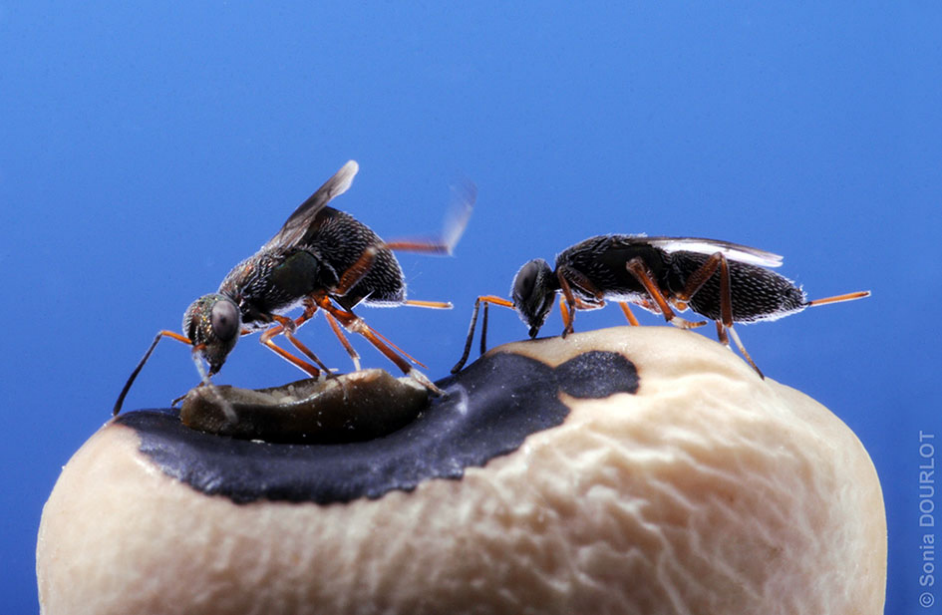
Non-injurious fighting behaviour: kicking in the eupelmid wasp *Eupelmus vuilleti* (photograph: Sonia Dourlot). (Online version in colour.)

Parasitoid contests may occur between adult female parasitoids and also between adult male parasitoids (e.g. [34]): in the interests of brevity we will consider only female–female interactions here. Contests typically arise when more than one foraging female encounters a host, or a patch of hosts. The first female to arrive may have assessed the quality of the host resource, invested in it via egg maturation, host-handling (including suppression via paralysis if the host is, for instance, a motile larva rather than an immotile egg) and oviposition before any other foragers arrive. Usually, females assess host quality and then either reject or accept the opportunity to oviposit before foraging for further hosts. However, females of the ichneumonid wasp *Hyposoter horticola* locate clusters of hosts that are not yet suitable for oviposition and return to monitor them repeatedly over several weeks until they are able to support parasitoid offspring [35,36]. It is typically disadvantageous for a female finding suitable hosts to share the resource with other females, because their offspring would compete, via scramble or contest competition, for the limited nutrition provided by each host. In some parasitoid species, females exploit the hosts they have found with little or no behavioural interactions and thus compete via their reproductive decisions and via the actions of their offspring. However, in the species considered here, an initial female may attempt to repel, via aggression, subsequently arriving females from the vicinity of the host. As the host resource also has value to later arriving females, which must access a host at some stage during their adult lives in order to reproduce, the initial female's attempts to repel them may be resisted and, indeed, they may attempt to drive the initial female away. In *H. horticola*, several females monitoring a given cluster of hosts may be present at the same time, leading to direct and aggressive interactions, especially when the hosts have developed to be suitable for oviposition. Females may fight intensely over a host egg cluster, involving the wasps ‘rolling with each other and poking each other with their ovipositor’ [35,36]. The details of how parasitoid females interact depend partially on their morphologies: some species have well-developed mandibles and can bite each other (sometimes fatally [37], see also [38]), and stingers that can inject venom (sometimes fatally [39]), while other species are relatively weaponless and fight via non-injurious behaviours such as pushing, mounting, kicking and chasing (e.g. the eupelmid wasp *Eupelmus vuilleti* [40–42], figure 1).

In the most likely case of interactions between one initial female and one later arriving female, the situation closely matches dyadic owner–intruder contests considered by classical game-theoretic models (e.g. [14]). These predict that contests will be won by individuals that have morphological (e.g. body size) or other physical (e.g. positional) advantages (greater resource-holding potential, RHP) and by individuals to which the resource has the higher value (*V*) and, further, that fatal fighting will evolve when the value of the current resource exceeds the expected value of future resources [38,43]. The observed behaviours of parasitoids largely comply with such predictions (reviewed in [21]). Although the most intuitively obvious prediction, that larger (stronger) females will win contests, is typically met (e.g. [44]), this is not always so and contest outcome and contest intensity (the degree of aggression exhibited) can be greatly affected by perhaps more subtle properties, such as subjective and objective resource value [35,40,41,44–47], and further considerations, such as the shared evolutionary interest of competitors, owing to close relatedness, and the timescale over which interactions are considered [48–50].

A number of recent studies have focused on the effects of a parasitoid's internal state on its contest performance and also there has been increased consideration of the influence of contest performance on a parasitoid's subsequent internal state. The nutritional status (energy reserves) of a parasitoid may influence its contest ability (RHP). Honey-fed *Goniozus* wasps have higher concentrations of haemolymph sugars, amino acids and lipids (assessed using mass spectrometry) than do starved wasps, but nutritional status did not affect the outcome of the dyadic contests studied by Snart *et al*. [51], possibly because an RHP-enhancing effect of feeding is matched by a higher value (*V*) being placed on winning by the starved wasps. Clearer results were found by Boisseau *et al*. [41], who assessed the metabolic costs of contest behaviours in *E. vuilleti* by measuring CO_2_ production using flow-through respirometry. Individuals with higher pre-contest metabolic rates tended to win contests for oviposition sites, which could be seen as a cost to maintaining high RHP. However, fighting for a host was less energetically costly than were the drilling behaviours involved in accessing weevil hosts and, as hosts are relatively common, it seems that these wasps benefit more from strategically waiting to re-use an oviposition hole drilled by another wasp than from contesting access to a given oviposition site. Titres of hormones that are associated with reproduction and social ranking in hymenopterans have also been assessed in *E. vuilleti*: post-contest titres of ecdysteroid were higher among females that had won than those that had lost, and losers had lower titres than females that had not experienced a contest [52]. Manipulative pre-contest injections of ecdysteroid (ecdysone) increased a female's probability of winning, without affecting their aggressiveness [52], and injecting juvenile hormone (JH III) increased aggressiveness, without affecting their probability of winning [53]. Further, clear effects of egg load asymmetries have been found in parasitoid contests: wasps with higher egg loads tend to win contests; higher egg load is interpreted as making a given host more valuable (increased *V*) to a wasp because it is better able to exploit it via oviposition ([40,45,47,54], see also [35]). Manipulative experiments that have injected ecdysteroid or juvenile hormone have found that both stimulate egg maturation and both increase a female's probability of winning a contest [52,53].

The experience of winning or losing a contest can affect the probability of an individual winning or losing subsequent contests. Typically, winners tend again to win and losers tend again to lose, possibly because the outcome leads to a reassessment of contest ability (RHP) by each individual. These prior-experience effects have been observed in laboratory experiments across numerous animal taxa, with loser effects typically stronger than winner effects [55]. An early model [56] predicted that a winner effect cannot exist without a loser effect: intuitively because if costs are too low to support a loser effect, then they are also low enough to support such a high initial perception of RHP that there is no advantage to raising it after a win. More than a decade later, this prediction remains ‘well-supported by the empirical literature’ ([57, p. 423]). Specifically, to date, several experiments have produced only a loser effect, and several have produced both a loser and a winner effect [55]; however, only one experiment, an initial study using the parasitoid *E. vuilleti*, has ever produced a winner effect without a loser effect [40]. It was suggested by Goubault & Decuignière [40] that winning wasps undergo physiological changes related to increased egg maturation that increase the subjective value (*V*) of the host to them whereas losers do not, especially since in this species contest outcomes are unrelated to size asymmetries (RHP): this possibility has not yet been explored using a game-theoretic model. In particular, it lies beyond the scope of Mesterton-Gibbons's model [56], which assumes winning to be equally valuable to both contestants. Further work, which explored the influence of *E. vuilleti* females’ experience of resource ability found that loser effects were not observable when females had not experienced hosts earlier (thus currently contested hosts would have high *V*) but were observable when a rich prior experience would have reduced the females' estimation of *V* for the contested hosts, and winner effects were not found at all [58]. Overall, it was concluded that winner and loser effects depend on the context in which the contest occurs, interacting with further factors that influence aggressiveness and the probability of winning.

These results exemplify a broader picture that has emerged from recent experiments and field studies [55, pp. 41–42]. It shows that prior-experience effects are highly context-dependent across several taxa in ways that existing theory does not address. Newer theory is needed to address conceptual issues that arise. For example, if two consecutive losses can increase an animal's probability of winning subsequently, as recently reported in olive fruit flies [59], then it seems insufficient to define a winner effect as an increased probability of winning after winning. No explanation has yet been offered for the observed experience effect, but future game-theoretic models will be central to the endeavour of testing proposed hypotheses.

## 4. Agro-ecological applications

There are several connections between parasitoid contests and agro-ecological applications. It is notable that many parasitoids function as beneficial natural enemies of crop pests and are deployed as biological control agents, contributing to sustainable food production. Owing to their small size, their field-biology is often not readily observable and their success in biocontrol is not always easy to predict. It has been recognized that inter- and intra-specific contest behaviours, which are relatively readily observable in the laboratory, can be used as indicators of whether given combinations of natural enemies are likely to coexist in the field and provide the desired biocontrol services [39,60–63]. Contest behaviour has also been used to consider how parasitoids may respond to the presence of invasive crop pests. Specifically, the scelionid wasp *Trissolcus basalis* preferentially oviposits into eggs of the brown marmorated stink bug, rather than its normal stink bug host. When developing in this novel host most of the wasp's offspring die but the few survivors are unusually large. As it was known that large adult *T. basalis* females are advantaged in contests for host access (high RHP), game-theoretic modelling was used to explore whether a size advantage might sustain a maternal preference for the invasive host and found that it could, provided that it acts in concert with advantages associated with prior host possession. This suggested that contests could mediate a more stable invasive pest–parasitoid association leading towards biocontrol of this rapidly spreading and extremely damaging pest [28].

Another example of an agro-ecological aspect to parasitoid contest research is that parasitoids foraging, and competing, for hosts in the modern-day field are unlikely to do so without also being exposed to one or more synthetically produced agro-chemicals. Sub-lethal doses of the insecticide pyriproxyfen, a juvenile hormone mimic that competes with juvenile hormone molecules on receptors, were found to stimulate egg production in *E. vuilleti* and also increase aggression during contests for host weevil larvae [42]. Given that pyriproxyfen is a juvenile hormonal mimic, and similar results have been found when dosing these wasps with juvenile hormone itself [53], the observed behavioural effect is not unexpected. However, similar effects on egg maturation and aggression have been observed in *Goniozus* wasps following sub-lethal doses of the neonicotinoid imidacloprid, which principally acts via disruption of neural signalling (S. Stothard & I.C.W. Hardy 2019, unpublished data). These agro-chemicals may be influencing parasitoid contests via a general stimulation of the wasp's metabolic rate (enhancing RHP) and/or by increasing egg loads (enhancing *V*), at least in the short term, but the longer-term consequences of such agro-chemical exposure remain to be evaluated.

## 5. Contests and parasitoid sociality

Given that some resource-contesting parasitoid species in the family Bethylidae exhibit these behaviours alongside sub-social maternal care and quasi-social brooding, bethylid contests and closely related phenomena have also been considered in regard to the influence of relatedness between contestants and the evolution of hymenopteran sociality, with the relatively low ‘levels’ of sociality exhibited by these parasitoids providing a useful contrast to many more frequently studied social and eusocial hymenopteran species, such as ants. (It should, however, be noted that bethylid sociality appears to be condition-dependent and does not involve reproductive altruism: as such, these wasp lineages are unlikely to be on an evolutionary trajectory towards the colonial superorganismality observed in ants [64].) Aggression during short-term contests between *Goniozus* females is exhibited at reduced levels when contestant females are siblings or perceive each other to be siblings [48]. Experimentally enforcing these normally single-foundress brooders into long-term multiple-foundress associations with a single host showed that foundress mortality was lower when hosts were larger (and resources were thus less limiting) and when foundresses were siblings [50]. Even though single foundresses achieve higher *per capita* production of adult offspring, inter-foundress relatedness and large host size may combine to reduce selection against communal reproduction. In *Sclerodermus*, single foundresses achieve little success when hosts are large, and thus *per capita* production of adult offspring is higher among multi-foundress groups ([65]; see also [66]).

Although cooperative reproduction has thus evolved in *Sclerodermus*, there is recent evidence for competitive, sometimes agonistic, interactions between females. Initially, during the host-attack phase, when females are commonly killed by the large and dangerous host, pairs of foundresses hold back from taking risks for longer when they are non-siblings rather siblings [67–69]. This scenario was explored using game theory by Mesterton-Gibbons & Hardy [70], who considered variation in the risks incurred to individual females, how the activities of the two females interact to increase the probability of success, and that the females may be relatives and thus share evolutionary interests. High values of all three properties are predicted to favour cooperation in host attack while for small values cooperation is not an ESS. Further, high relatedness is predicted to be required for cooperation to evolve, but it may be subsequently maintained among non-relatives, as long as the actions of the females interact to effect success, and the probability of being killed by the host during attack is modest.

Subsequently, if hosts are successfully suppressed, *Sclerodermus* co-foundresses may cooperatively tend the communally produced brood, but also exhibit differential reproduction (reproductive skew) according to body size asymmetries and temporal priority [71], with directly aggressive, and sometimes fatal, contest interactions between females observed [37]. Further, foundresses appear to be competing over the sex ratios that they contribute to the communal broods, via pre- or post-ovipositional dominance and/or infanticide [66,71], as explored by recent game-theoretic modelling [72]. Overall, the quasi-sociality exhibited by *Sclerodermus* is cooperative to a degree but is also condition-dependent and involves conflicts of interest, competition and contests.

## 6. Conclusion

The ground-breaking development of ESS-based animal contest research around 50 years ago and the vast body of research into parasitoid evolution and ecology were not strongly connected until approximately 25 years ago. These fields have become increasingly, and beneficially, connected, improving the understanding of parasitoid behaviour in both fundamental and applied contexts, with reciprocal benefits to the general field of evolutionary behavioural ecology.

## Data Availability

There are no data directly associated with this article.
